# Report from the 28th Meeting on Toxinology, “Toxins: What’s up, Doc?”, Organized by the French Society of Toxinology on 28–29 November 2022

**DOI:** 10.3390/toxins15020126

**Published:** 2023-02-04

**Authors:** Pascale Marchot, Évelyne Benoit, Ziad Fajloun, Sylvie Diochot

**Affiliations:** 1 Laboratoire Architecture et Fonction des Macromolécules Biologiques (AFMB), Faculté des Sciences—Campus Luminy, CNRS/Aix-Marseille Université, F-13288 Marseille, France; 2 Service d’Ingénierie Moléculaire pour la Santé (SIMoS), Département Médicaments et Technologies pour la Santé (DMTS), Institut des Sciences du Vivant Frédéric Joliot, Université Paris-Saclay, CEA, INRAE, ERL CNRS 9004, F-91191 Gif-sur-Yvette, France; 3 Laboratory of Applied Biotechnology (LBA3B), AZM Center for Research in Biotechnology and Its Applications, Doctoral School for Sciences and Technology, Lebanese University, Tripoli 1300, Lebanon; 4 Department of Biology, Faculty of Sciences 3, Campus Michel Slayman Ras Maska, Lebanese University, Tripoli 1352, Lebanon; 5 Institut de Pharmacologie Moléculaire et Cellulaire, Université Côte d’Azur, CNRS, Sophia Antipolis, F-06560 Valbonne, France

**Keywords:** animal toxin, bacterial toxin, deep learning, marine toxin, medical application, neuromuscular junction, plant toxin, toxin structure–function relationship, toxinomics, toxin receptor–target, toxin structure, toxin tracking

## Abstract

The French Society of Toxinology (SFET) organized its 28th annual meeting on 28–29 November 2022 (RT28). The central theme of this meeting was “Toxins: What’s up, Doc?”, emphasizing the latest findings on animal, bacterial, algal, plant and fungal toxins through sessions dedicated to deep learning, toxin tracking and toxinomic advances, shared by ca. 80 participants. The abstracts of the 10 invited and 11 selected lectures and 15 posters, along with the names of the Best Oral Communication and Best Poster awardees, are presented in this report.

## 1. Acknowledgments

**Figure 1:**
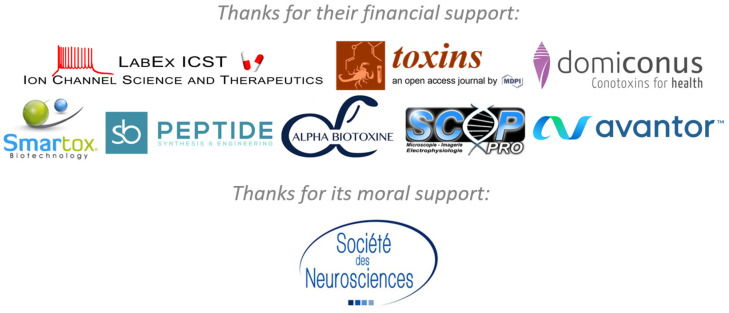
Sponsors’ logos.

We warmly acknowledge the contribution of all the people who work daily at ensuring the national and international shinning of the French Society of Toxinology (SFET) and those who made the 28th Meeting on Toxinology a success. We also address special thanks to our sponsors who, this year again, supported our meeting (Figure 1).

## 2. Preface

The 28th edition of the annual Meetings on Toxinology (RT28) of the French Society of Toxinology (SFET, http://sfet.asso.fr/international (accessed since 1 September 2022)) was held at the Institut Pasteur of Paris on 28–29 November 2022.

Recent progress in modern toxinology themes related to deep learning, toxin tracking and toxinomics and in the more traditional theme neuromuscular junctions were presented by 10 experts in the field from seven countries (Austria, Germany, Norway, Portugal, Spain, the United States of America and France). In fact, 38% of the ca. 80 participants were foreigners, which once again highlights the international attractiveness of these Meetings on Toxinology held by the SFET.

Eleven other speakers, selected among researchers, postdoctoral fellows and students, also presented their work in these sessions or in the latest session traditionally dedicated to recent, albeit out-of-theme studies. Fifteen posters were also displayed and discussed all throughout the meeting, of which 11 were presented as 3 min flash-talks.

The contribution of artificial intelligence in the search for new drugs or of bioinformatics models in genomic or structural studies has been discussed. Two conferences concerned the use of bacterial toxins to understand the pathophysiology of the neuromuscular junction. Marine toxins and phycotoxins were in the spotlight for the biosensing aspects concerning public health problems. The progress in toxinomics was presented for the monitoring of the evolution of ant venoms, marine cone venoms, and marine cyanotoxins and phycotoxins. Finally, the exploration of the toxicity of snake venom, the mode of action of animal or bacterial toxins, and the recent progress in protein engineering to create safer antivenoms completed this 28th Meeting on Toxinology.

**Figure 2:**
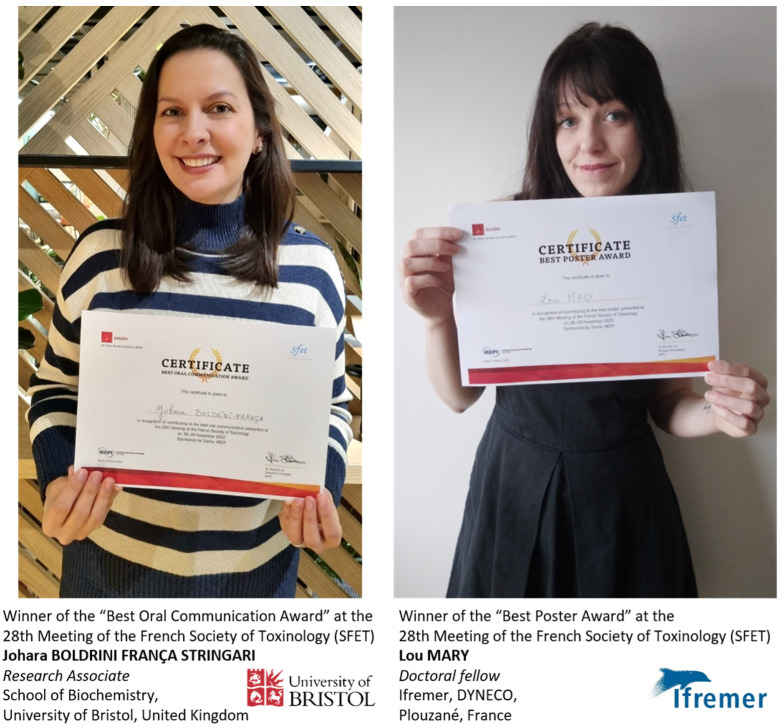
The “Best Oral Communication” and “Best Poster” Awardees at the 28th Meeting of the French Society of Toxinology (SFET).

Owing to a donation from the MDPI journal *Toxins*, two prizes of EUR 300 each were awarded to the best oral communication and the best poster (Figure 2), both selected via a vote by a jury comprising 8 of the 10 invited speakers (Mònica Campàs, Alan R. Hauser, Ivan Koludarov, Doris Marko, Jean Louis Marty, Manuel J. Tenorio, Eivind A. B. Undheim and Vitor Vasconcelos) and 11 of the 12 members of the SFET board (Évelyne Benoit, Katrina Campbell, Alexandre Chenal, Sylvie Diochot, Sébastien Dutertre, Ziad Fajloun, Daniel Ladant, Christian Legros, Pascale Marchot, Michel R. Popoff and Loïc Quinton).

Last but not least, we warmly thank the Editors of *Toxins* for supporting the publication of a Special Issue also entitled “Toxins: What’s up, Doc?” and for gathering the materials used in this meeting report, along with peer-reviewed original articles and reviews. We are confident that this Special Issue will be attractive to all, including those of our colleagues who could not attend the RT28 meeting, and that it will represent a comprehensive source of information for researchers and students in toxinology.

## 3. Scientific and Organizing Committee (SFET Board of Directors)

Évelyne Benoit, CEA de Saclay, Gif-sur-Yvette, France
Katrina Campbell, Institute for Global Food Security, Queen’s University Belfast, Belfast, Northern Ireland
Alexandre Chenal, Institut Pasteur, Paris, France
Sylvie Diochot, Institut de Pharmacologie Moléculaire et Cellulaire, Valbonne, France
Sébastien Dutertre, Institut des Biomolécules Max Mousseron, Montpellier, France
Ziad Fajloun, Doctoral School of Science and Technology, Lebanese University, Tripoli, Lebanon
Daniel Ladant, Institut Pasteur, Paris, France
Christian Legros, Université d’Angers, Angers, France
Pascale Marchot, CNRS/Aix-Marseille Université, Marseille, France
Michel R. Popoff, retired from Institut Pasteur, Paris, France
Loïc Quinton, Université de Liège, Liège, Belgium
Michel Ronjat, retired from Institut du thorax, Université de Nantes, Nantes, France

## 4. Invited Lectures

### 4.1. Artificial Intelligence and the Revolution of the Intelligent Drug

MoingeonPhilippe*Research and Development, Servier, Suresnes, France.*Correspondence: philippe.moingeon@servier.com

**Abstract:** Artificial intelligence (AI) encompasses technologies recapitulating four dimensions of human intelligence, i.e., sensing, thinking, acting and learning. The convergence of technological advances in those fields allows us to integrate massive data and build probabilistic models of the reality of a problem. The latter can be continuously updated by incorporating new data to inform decision-making and help predicting the future. In support of drug discovery and development, AI allows us to generate disease models using data obtained by extensive molecular profiling of patients. Given its superior computational power, AI can integrate those big multimodal data to generate models allowing us to (i) represent patient heterogeneity and (ii) identify therapeutic targets with inferences of causality in the pathophysiology. Additional computational analyses can help identifying and optimizing a drug interacting with this target, or even repurposing an existing molecule for a new indication. AI-based modelling further supports the identification of biomarkers of efficacy, the selection of appropriate combination therapies and the design of innovative clinical studies with virtual placebo groups. The convergence of biotechnologies and AI is fostering the emergence of a computational precision medicine capable in the future to propose therapies or preventive measures precisely tailored to patient characteristics in terms of their physiology, disease features and environmental risk exposure. 

**Keywords:** artificial intelligence; computational precision medicine; disease model; drug development

### 4.2. Comparative Genomic Approaches to Identify Novel Virulence Determinants

HauserAlan R.^1^,^2^*AllenJonathan^3^OzerEgon^2^PincusNathan^1^1Department of Microbiology/Immunology, Northwestern University, Chicago, Illinois, USA.2Department of Medicine, Northwestern University, Chicago, Illinois, USA.3Department of Microbiology/Immunology, Loyola University Chicago Stritch School of Medicine, Maywood, Illinois, USA.*Correspondence: ahauser@northwestern.edu

**Abstract:** A fascinating aspect of microbiology is the large amount of genetic diversity between bacterial species, which is responsible for the myriad of pathogenic strategies they employ to cause strikingly different infections. However, only recently have technological advances uncovered a wealth of strain-to-strain genetic diversity within individual bacterial species. This diversity manifests as single nucleotide variants (SNVs), insertion-deletions, and large accessory genomes. Together, these differences confer strain-specific pathogenic traits that have important disease implications. Several comparative genomic strategies have been developed and optimized to interrogate bacterial genomes for differences that impact infection. Many of these strategies have several steps in common, including the collection of a population of isolates, sequencing of the isolates, quantification of the virulence of the isolates, and the use of biostatistical approaches (such as genome-wide association studies or machine learning) to associate genomic features with virulence phenotypes. Genomic features identified by these strategies may then be confirmed using a second cohort of isolates or by genetic testing. Here, we provide a brief overview of these strategies and illustrate their power with the following previously published example. The accessory genomes of 100 *Pseudomonas aeruginosa* bloodstream isolates were determined by whole-genome sequencing. The virulence of each isolate was quantified by determining its 50% lethal dose in a mouse bacteremia model. Accessory genomic elements associated with highly virulent isolates were identified, and 15 were assessed for a causal role in virulence by the testing of deletion mutants. Deletion of 11 of the 15 accessory genomic elements resulted in decreased virulence in the mouse model, whereas deletion of 5 control accessory genomic isolates did not. One accessory genomic element, AGEv15, was further characterized and shown to encode a contact-dependent growth inhibition system that functioned as a virulence factor in cell culture and mouse models of infection. As larger populations of bacteria are routinely sequenced, comparative genomic approaches will play an increasing role in enhancing our understanding of intraspecies differences in virulence.

**Keywords:** bacterial genome; bacterial virulence; *Pseudomonas aeruginosa*

### 4.3. Protein Language Models Help Investigating Structure-Function Relationship and Evolution of Toxins

KoludarovIvan*BurkhardRostTechnical University of Munich (TUM), Department of Informatics, Bioinformatics & Computational Biology, Justus Liebig University of Giessen, Munich, Germany.*Correspondence: atjcoludar@gmail.com

**Abstract:** Modern biology has accumulated a unique wealth of unannotated data arguably capturing more detail than the fundamental theory. Many experts have been acknowledging this in their call for an extended synthesis of evolutionary theory. The core question of how organisms evolve new traits and functions is ripe for theory advancement, with the modern wealth of information including millions of genes and proteins whose structure and function can be modelled with AI. One of the promising developments comes from natural language processing models, which were repurposed to treat amino acids as individual words and proteins as sentences. In our research, protein Language Models (pLMs) differentiated the major molecular and functional forms of several toxin families that have been identified by decades of structure-function research and allowed us to go further and propose scenarios for their evolution. Using embeddings from pLMs appears to represent a progress toward predicting molecular activity from sequence data by analogy with prior knowledge and allows us to annotate new sequences extracted from the genomic data with greater confidence than possible just using phylogenies. We suggest that already existing and future applications of pLMs will allow us to make more sense of existing unannotated data and get much closer to answering a question what facilitates a change of function that accompanies every toxin evolution, as well as pinpoint changes of function and activity within toxin families.

**Keywords:** evolution of function; protein Language Models; toxin family

### 4.4. Tracking Emerging Mycotoxins in Food: Development of an LC-MS/MS Method for Free and Modified Alternaria Toxins

MarkoDoris*Department of Food Chemistry and Toxicology, Faculty of Chemistry, University of Vienna, Waehringerstrasse 38, 1090 Vienna, Austria.*Correspondence: doris.marko@univie.ac.at

**Abstract:***Alternaria* toxins are formed by black molds of the genus *Alternaria*, which occur ubiquitously and are able to grow under varying temperature and moisture condition as well as on a large diversity of substrates. Reports on the occurrence of *Alternaria* toxins comprise a broad spectrum of plant-based food commodities including grain and grain-based products, apples, oilseeds, sunflower oil and tomato products. Depending on the respective *Alternaria* strain, substrate and growth conditions, the spectrum and pattern of formed secondary metabolites can differ substantially. So far, known *Alternaria* metabolites can be assigned to five substance classes: dibenzo-α-pyrones (e.g., alternariol, AOH), perylene quinones (e.g., altertoxin II), tetramic acid derivatives (e.g., tenuazonic acid, TeA), aminopentol esters (AME) and miscellaneous structures (e.g., tentoxin, TEN). Recent studies demonstrated, that metabolism of plants might even increase the spectrum of compounds potentially present in food. We developed validated LC-MS/MS methods for assessment of free and modified *Alternaria* toxins in tomato products, sunflower seed oil and wheat flour. In April 2022, the European Commission published a recommendation of indicative values for AOH, AME and TeA in certain food commodities [1]. However, *Alternaria alternata* is able to generate a broad spectrum of secondary metabolites with different activity profile. Thus, in native toxin mixtures, a complex overlay of biological activities might occur. But already for single toxins, the concentration range is decisive for the toxic effect. In low micromolar concentrations, AOH and its monomethyl ether AME possess immunosuppressive and endocrine disruptive properties. At higher concentrations, genotoxic and weak mutagenic potential can be observed, whereby genotoxicity arises predominantly from interference with topoisomerase. Besides TeA, TEN and the two major dibenzo-α-pyrones AOH and AME, *Alternaria* spp. may produce significant amounts of perylene quinone derivatives, such as alterperylenol (ALP), altertoxins (ATX) I-III and stemphyltoxin III (STTX-III). ATX-II, exceeds by far the genotoxicity of AOH, and represents an important DNA-damaging component in complex mixtures. So far, the occurrence of perylene quinones in food is still unknown. Taken together, *Alternaria* toxins comprise a spectrum of mycotoxins with different molecular targets. Data are accumulating, arguing for relevance of these compounds in the food chain.

**Keywords:** altertoxin; alternarial; LC-MS/MS; tenuazonic acid

### 4.5. Biosensors and Other Biotechnological Tools for Tracking Marine Toxins

CampàsMònica*Marine and Continental Waters (AMiC), Institut de Recerca i Tecnologia Agroalimentàries (IRTA), La Ràpita, Spain.*Correspondence: monica.campas@irta.cat

**Abstract:** Biosensors and other biotechnological tools can contribute to guarantee the sustainable management of oceans and seas. The high sensitivities, low limits of detection, short analysis times, simplicity of use, low cost, miniaturisation, automation and portability make interesting their implementation in seafood safety and public health protection programmes. The deployment of these tools in the marine environment can contribute to manage shellfish production areas, to understand phytoplankton dynamics, to study toxin transfer within food webs, to identify areas at risk, to predict the state of coastal waters, and to evaluate impacts of the climate change, among others. In the Marine and Continental Waters Program of IRTA, we develop biotechnological tools for the detection of analytes of interest in aquaculture, fisheries, marine and environmental monitoring. Self-assembling immobilisation strategies, use of magnetic particles as supports, isothermal DNA amplification techniques, miniaturised electrodes and multiplexing detection capabilities are examples of our recent efforts. The biotechnological tools have been applied to the analysis of shellfish, fish, microalgae, seawater and even human body fluids. Our latest works will be reviewed and the new challenges to tackle will be summarised.

**Keywords:** biosensor; environmental monitoring; food safety

### 4.6. Biosensors for the Detection of Toxins: A Review on Selection of Bioreceptor

MartyJean Louis*Université de Perpignan Via Domitia, 52 avenue Paul Alduy, 66860 Perpignan Cedex.*Correspondence: sensbiotech@gmail.com

**Abstract:** The global success of the glucose biosensor has led many scientists to anticipate the development of an ideal biosensor with exceptional characteristics. Despite of much progress at the research level, the biosensors market has not experienced the expected outcome mainly due to difficulties in obtaining stable, specific and sensitive bioreceptor. The present talk aims to discuss all the possible alternatives to conventional biomolecules employed in the design of biosensors. Recent advancement in modified enzymes, nanozymes, nanobodies, aptamers, peptides, membrane-imprinting polymers, DNazymes and protein scaffolds will be highlighted. These recognition elements are considered to be major players in the future biosensor architectures.

Despite good binding affinities, antibodies are characterized with certain limitations. In this context, recombinant antibody fragments, Fab, Fv (variable fragment), scFv (single chain variable fragment) and VH containing all specific recognition sites of the antibodies, have been explored for the fabrication of immunosensors. Recently, heavy chain antibodies (hcAbs) have gained considerable attention due to their unique structure, being composed of only heavy chains. They are also known as nanobodies due to their small size. Another class of compounds, called aptamers, encompasses short sequences of artificial DNA and RNA. Like antibodies they display strong affinities for their targets with dissociation constants close to the nano-picomolar. A multitude of aptamers have been developed and integrated in aptasensors development for the detection of a wide range of target analytes. Other biosensing formats are based on the ribozymes and DNAzymes as biorecognition elements. With the development of combinatorial protein engineering and selection techniques, it has become possible to design artificial affinity proteins with the desired properties. These proteins, termed alternative scaffold proteins, are most often chosen for their stability, low immunogenicity, ease of engineering. They have various names: affibodies, alphabodies, affitins, atrimers… In parallel to biology-based receptors for toxins and small molecules, researchers are also interested in the potential of synthetic materials to achieve selective recognition of toxins. Molecularly Imprinted Polymers (MIPs) offer a method of creating substrate-specific materials, and subsequently, Peptide nucleic acids (PNA) can be used for the analysis of DNA. If the first biosensor was an enzyme sensor, the future can also witness an enzyme sensor based on artificial enzyme biomimetic nanomaterial. They offer the advantages of low cost, high stability, easy of production and tunability of the catalytic activity.

**Keywords:** bioreceptors; biosensors; MIP

### 4.7. Ant Venom Evolution: Insights from the Genetically Hyper-Diverse Greenhead Ant

UndheimEivind A. B.*Centre for Ecological and Evolutionary Synthesis, Department of Biosciences, University of Oslo, Oslo, Norway.*Correspondence: e.a.b.undheim@ibv.uio.no

**Abstract:** Ant venoms have emerged as rich sources of extremely diverse peptide toxins, contrasting previously widespread misconceptions that they are primarily acid-based. While this diversity makes ant toxins attractive as pharmacological tools, the eusocial lifestyle of ants also provides opportunities for studying how venom evolution may be affected by selection at different levels of biological complexity. In this talk, I will present some of our recent work on the composition and function of ant venoms and their toxins, with a focus on how colony structure and colony-level selection may drive their evolution. 

**Keywords:** ant venom; evolution; peptide; selection

### 4.8. OMIC’s Approaches on Phycotoxins and Cyanotoxins

VasconcelosVitor^1^,^2^*1CIIMAR—Interdisciplinary Center of Marine and Environmental Research, Terminal de Cruzeiros do porto de Leixões, Av general Norton de Matos, 4450-208 Matosinhos, Portugal.2Faculty of Sciences, University of Porto, Rua do campo Alegre, 4069-007 Porto, Portugal.*Correspondence: vmvascon@fc.up.pt

**Abstract:** Phycotoxins and cyanotoxins produced by microalgae and bacteria cause severe problems with large impacts in terms of ecosystem health, in human health and in the blueeconomy. Recently, genomics provides valuable early warning tools to evaluate the risk of occurrence of the toxin-producing organism as well as the potential of toxins occurrence. Genomics is also fundamental as a tool in a polyphasic approach to unravel new biodiversity, to perform phylogenetic studies on species distribution as well as to define risks of consumption of algae and cyanobacteria based food and feed. Omics applications have permitted to determine the incidence and prevalence of toxin producing genotypes in natural populations via metagenomics studies and the potential for toxin occurrence via the study of the transcriptome. Novel toxins and other bioactive molecules are being unraveled via metabolomics approaches and their mechanisms of action discovered using advanced proteomics techniques. Heterologous expression of gene clusters responsible for the production of cyanobacteria bioactive metabolites allows us to use these tools for biotechnological applications. In this talk, we will approach omics tools in current cyanotoxin and phycotoxin research and technological applications and the future needs to use them in monitoring as well as in quality control of products. 

**Keywords:** cyanotoxin; omics; phycotoxin

### 4.9. Venom Gland Evolution and Conotoxin Diversification in Cone Snails

DutertreSebastien*IBMM, Univ Montpellier, CNRS, ENSCM, Montpellier, France.*Correspondence: sebastien.dutertre@umontpellier.fr

**Abstract:** Many animals produce toxic secretions to facilitate their survival, and the toxins and their mechanisms of expression are influenced by ecological interactions with prey and predators. Some cone species are capable of deploying different venoms depending on the defensive or predation intent. These compositionally distinct venoms are produced from a single gland and injected using the same envenoming apparatus, but the toxins are differentially expressed from other sections of the venom duct. Based on the analysis of the proximal section (near the bulb) and the distal section (near the proboscis) of different species of cones, several cases seem to be emerging. For example, the fish-eating species *Conus geographus* has a gland divided into two roughly equal parts, each producing specific toxins. Thus, the injection of toxins from the distal part is triggered by a predation stimulus, while the toxins from the proximal part are deployed for defensive purposes. This strategy also exists in some more recently divergent vermivorous species such as *Conus vexillum*, whose defensive venom toxins dominate the proximal duct. On the other hand, in other species such as *Conus planorbis*, the defensive toxins are not limited to the proximal section, with a more diffuse production of toxins along the duct. Consistent with its reminiscent ancestral nature, the venom gland of *Conus distans* lacks distinct compartmentalization, and each section produces a similar toxin composition. Finally, it would seem that in some molluscivorous species, the sections of the gland producing predation and defensive venoms are reversed compared to *Conus geographus*. These comparisons support the hypothesis that venom gland compartmentalization arose in early worm-eating species and facilitated the evolution to molluscivorous and piscivorous diets.

**Keywords:** conotoxin; defense; evolution; predation; venom gland

### 4.10. Adventures in West Africa: Venomics of Eastern Atlantic Cone Snails

TenorioManuel J.^1^*AbaldeSamuel^2^,^3^GalindoJuan Carlos G.^4^Pardos-BlasJosé Ramón^2^ZardoyaRafael^2^1Departamento de CMIM y Química Inorgánica-INBIO, Facultad de Ciencias, Universidad de Cádiz, Puerto Real, Cádiz, Spain.2Departamento de Biodiversidad y Biología Evolutiva, Museo Nacional de Ciencias Naturales (MNCN-CSIC), Madrid, Spain.3Department of Zoology, Swedish Museum of Natural History, Stockholm, Sweden.4Departamento de Química Orgánica-INBIO, Facultad de Ciencias, Universidad de Cádiz, Puerto Real, Cádiz, Spain.*Correspondence: manuel.tenorio@uca.es

**Abstract:** With more than 900 species known, cone snails are a highly diverse natural group living referentially in the intertidal zone of tropical and subtropical regions worldwide. They are key marine predators that produce venom to prey on worms, snails, and fish, as well as to defend against predators. The venom is composed of hundreds of peptides named conotoxins. There is a very active ongoing research on conotoxins, mostly focused on Indo-Pacific fish- and mollusk-eating species, whereas a large majority of mostly vermivorous cone snail species from other geographic areas have been traditionally neglected. For about 10 years now, we have been systematically sampling and studying the biodiversity of the cone snails from the Mediterranean area and West Africa. This area can be considered as one important hotspot of biodiversity for cone snails, particularly the Cape Verde Islands, but also Senegal and Angola. Most of the species studied are vermivorous, with the notable exception of a piscivorous one, *Chelyconus ermineus*. We determined the transcriptome of the venom duct of three individuals of this species. Its venom repertoire includes at least 378 conotoxin precursors, which could be ascribed to 33 known and 22 new (unassigned) protein superfamilies, respectively. Considerable intraspecific variation was observed, as each individual had many exclusive conotoxin precursors, and only 20% of all inferred mature peptides were common to all individuals. Additionally, we sequenced the transcriptomes of the venom glands of closely related species of vermivorous cones endemic to West Africa from genera *Africonus* and *Varioconus*. Venom repertoires were compared within a phylogenetic framework using *Kalloconus* species as outgroup. The total number of conotoxin precursors per species varied between 108 and 221. A common set of four conotoxin precursor superfamilies (T, O1, O2 and M) was expanded in all studied cone species. They are considered the basic venom toolkit for hunting and defense in the West African vermivorous cone snails. Moving forward, we determined the chromosome-level genome of the venomous Mediterranean cone snail *Lautoconus ventricosus*. On the basis of venom gland transcriptomes, we annotated 262 complete genes encoding conotoxin precursors, hormones, and other venom-related proteins. These genes were scattered in the different pseudochromosomes and located within repetitive regions. This genome becomes a reference for assembling and analyzing new cone snail genomes. Finally, we have used proteomic techniques for validating the conotoxins and other venom peptide sequences resulting from our transcriptomic and genomic analyses. 

**Keywords:** cone snail; conotoxin; proteomics; transcriptomics

## 5. Oral Presentations

### 5.1. Neuromuscular Junction and Brainstem Nuclei Are the Targets of Tetanus Neurotoxin in Cephalic Tetanus

FabrisFederico^1^VaraniStefano^1^TonellatoMarika^1^MatakIvica^2^ŠostaricPetra^2^MeglicPatrik^2^CaleocMatteo^1^MegighianAram^1^,^3^RossettoOrnella^1^,^4^,^5^MontecuccoCesare^1^,^4^PirazziniMarco^1^,^5^*1Department of Biomedical Sciences, University of Padova, Via Ugo Bassi 58/B, 35131 Padova, Italy.2Department of Pharmacology, School of Medicine, University of Zagreb, Šalata 11, 10000 Zagreb, Croatia.3Padova Neuroscience Center, University of Padova, Via Giuseppe Orus 2, 35131 Padova, Italy.4Institute of Neuroscience, Italian Research Council, University of Padova, Via Ugo Bassi 58/B, 35131 Padova, Italy.5Interdepartmental Research Center of Myology CIR-Myo, University of Padova, Via Ugo Bassi 58/B, 35131 Padova, Italy.*Correspondence: marco.pirazzini@unipd.it

**Abstract:** Cephalic tetanus (CT) is a very dangerous form of tetanus characterized by simultaneous cerebral palsy and spastic paralyses following the intoxication of cranial nerves by tetanus neurotoxin (TeNT). We established a CT mouse model by locally injecting TeNT into the whisker pad (WP) and then examined both the neuromuscular activity through live imaging and electromyography and analyzed toxin activity in specific central and peripheral synapses. We report the unexpected finding that TeNT cleaves its target VAMP within facial neuromuscular junctions (NMJ) causing a flaccid paralysis reminiscent of botulism. This overshadows the canonical activity of TeNT in the brainstem, which however rapidly spreads throughout neuronal nuclei controlling functions essential for survival, including respiration. Such a toxin movement occur via an intraparenchymal process of rapid toxin dissemination, which may uncover a still unknown method of TeNT movement in the nervous system. Our findings suggest to inoculate anti-TeNT immunoglobulins in any case of idiopathic facial palsy, especially if associated with face skin wounding: in case of tetanus, this innocuous treatment might protect patients from the nefarious consequences of a rapidly developing form of the disease.

**Keywords:** cephalic tetanus; facial palsy; neuromuscular junction; spastic paralysis; tetanus neurotoxin

### 5.2. Botulinum Toxin Type A Duration Enhancement by Mu-Conotoxin CnIIIC

MachicoaneMickaël^1^*OnillonPaul^1^StaziMarco^2^TonellatoMarika^2^CrosCécile^1^RossettoOrnella^2^PirazziniMarco^2^DoussalJean-Marc Le^1^1Fastox Pharma SA, Lausanne, Switzerland.2Department of Biomedical Sciences, University of Padova, Padova, Italy.*Correspondence: machicoane@fastox.ch

**Abstract:** Notwithstanding the great success of Botulinum Neurotoxin (BoNT) use in therapy and aesthetic medicine, there is still room to improve BoNT pharmacological performance, especially by accelerating the onset of action and extending its duration of action. In order to fill this gap, we tested whether the onset of BoNT/A pharmacological action can be accelerated by combining toxin injection with a fast-acting inhibitor of muscle contraction, i.e., conotoxin CnIIIC.

The combination of BoNT/A with CnIIIC showed a clear acceleration in the onset of myorelaxant effects compared to BoNT/A alone, as evaluated with the DAS model. Intriguingly, we observed an unexpected potentiation of BoNT/A activity also at later timepoints, when CnIIIC has no longer effects when injected alone. To figure out the mechanism behind the synergy, we monitored the time course of cleaved-SNAP25 at the neuromuscular junction: the signal could be detected after 3 h when BoNT/A was co-injected with CnIIIC, compared to 9 h when BoNT/A was injected alone, supporting an accelerated accumulation of cl-SNAP25. To understand the biological process behind such a faster entry of BoNT/A, we evaluated the effects of CnIIIC alone on neurotransmission by monitoring the m-EPP at the NMJ, i.e., the spontaneous vesicle fusion events reflecting motoneuron excitability. We found that a few hours after injection CnIIIC strongly increased the frequency of m-EPP, which normally are instead relatively rare.

Altogether, we hypothesize that this synergistic effect is induced by the rapid muscle blockade caused by CnIIIC, which triggers an immediate potentiation of motoneuron excitability via a still unknown retrograde signal. Considering that BoNT/A enters via synaptic vesicle recycling, this in turns favors and accelerates BoNT/A uptake, and thus activity. Our findings uncovered the crosstalk between muscle and motoneuron at the NMJ as a novel layer to potentiate BoNT/A pharmacological performance.

**Keywords:** botulinum toxin type a; conotoxin; neuromuscular junction

### 5.3. Toxins to Fight Cancers: Developing a New Radiotracer from Snake Toxin Derivatives, Mambaquaretins

PetrovicGoran Stanajic^1^*KesslerPascal^1^GillesNicolas^1^TruilletCharles^2^ServentDenis^1^1Université Paris-Saclay, CEA de Saclay, Joliot, DMTS, SIMoS, EMR CNRS/CEA 9004, Gif-sur-Yvette, France.2BioMaps, Service Hospitalier Frédéric Joliot, CEA, Orsay, France.*Correspondence: goran.stanajicpetrovic@cea.fr

**Abstract:** Cancers represent one of the biggest challenges for modern medicine. Suffering from a lack of specificity as well as a way-too-important delay in their diagnosis, patient survival rates would be improved by a personalised management based among other things on the tumour biochemical signature. This signature may take the form of a metabolic specificity, or the ectopical expression of a specific receptor, allowing the establishment of an improved therapeutic strategy with better statistics. In order to do so, signature specific detection agents, allowing an early detection of those abnormal events with great precision, are mandatory. Mambaquaretin is on the verge of becoming one of those very agents. This peptidic toxin is the first molecule to have both a tremendous affinity and selectivity for the type-2 vasopressin receptor, or V2R, from the GPCR family. This family is of an extreme diversity and 70% of our actual pharmacopoeia is directed against one of its members. Alas, they are, in the same way as ion channels, amply underexploited as markers in the clinical diagnosis and/or imagery fields. V2R seems to be ectopically expressed by several cancer types including but not restricted to kidney, breast, prostate and lung cancers. Some of them still do not profit from a satisfying therapeutic support. Mambaquaretin thus shows all the characteristics of an imagery diagnosis agent as a radioactive tracer. Without either acute or chronic toxicity and with a pharmacokinetic suitable for such an application, the 89 zirconium-labelled molecule is in trial so as to make it a robust, reliable and clinic-ready marker. Its engineering led to the development of an improved derivative named MQ-LEAD with an even greater affinity for human V2R. Its pharmacokinetic profile still has to be evaluated, but the preliminary data are engaging. MQ-LEAD: the new Swiss army knife of modern medicine?

**Keywords:** cancer; radiotracer; toxin

### 5.4. Development of an Innovative Methodology of Antivenomics Approach, Combining the Use of Magnetic Beads and Mass Spectrometry

RedureauDamien^1^CrassetThomas^1^AmorimFernanda Gobbi^1^MenziesStefanie^2^MazzucchelliGabriel^1^CasewellNicholas^2^QuintonLoïc^1^*1Mass Spectrometry Laboratory, MolSys RU, University of Liège, 4000 Liège, Belgium.2Centre for Snakebite Research and Interventions, Tropical Disease Biology Department, Liverpool School of Tropical Medicine, Pembroke Place, Liverpool, L3 5QA, UK.*Correspondence: loic.quinton@uliege.be

**Abstract:** Snakebite is classified as a Category A neglected tropical disease by the WHO, as it causes the death of about 150,000 people every year, mostly in rural and poor areas of the world. Snake envenoming is classically treated by injecting antivenom, which is made of antitoxin antibodies (Igs) collected from immunized animals. However, these treatments may induce immunological reactions including severe adverse reaction to the patient. Moreover, venom compositions strongly differ from species, gender and habitat, explaining why providing broadly effective antivenoms is a real challenge. In this context, quantitatively evaluating the toxin-binding capability of any antivenom is crucial to improve the production of effective snakebite therapeutics. In this study, we propose an alternative methodology for the so-called “antivenomics” methodology. Indeed, affinity columns coupled to mass spectrometry have been demonstrated performant, but their preparation and their lifetime represent constraints to the throughput of antivenom evaluation. In this work, we exploit the potential of magnetic beads, LC-MS and shotgun proteomics mass spectrometry to speed up antivenom efficacy characterization. The antivenom Igs are bound to magnetic beads, before being incubated in the presence of various venoms. Comparative MS analysis of the toxins remaining in suspension (not recognized by Ig) and those remaining on the beads (recognized by Ig) allows the binding selectivity of the antivenom to be determined. The strategy is demonstrated here with venom from the medically important African viper, *Echis ocellatus*.

**Keywords:** antivenomics; magnetic beads; mass spectrometry; venom

### 5.5. Toxin Tracking—Which Detection Method Is the Most Appropriate?

CampbellKatrina*Institute for Global Food Security, School of Biological Sciences, Queen’s University Belfast, Belfast, North Ireland.*Correspondence: katrina.campbell@qub.ac.uk

**Abstract:** Contaminant monitoring from microbiological, chemical and fraudulent sources in agri-food production is an important yet complex issue. A huge investment in time and effort is placed on these activities by regulatory and industrial laboratories. Although sophisticated techniques such as chromatography and spectrometry provide accurate and conclusive results, screening tests allow a much higher low cost throughput of samples with less operator training. Biosensors combine a biological recognition element with a transducer to produce a measurable signal proportional to the extent of interaction between the recognition element and contaminant. 

The different uses of biosensing instrumentation available are extremely varied, with agri-food analysis illustrating emerging applications. However, ELISA and lateral flow tests dominate this market as difficulties remain in combining sample preparation for feed/food contaminant analysis with biosensor technology for point of site testing. Nonetheless, the advantages offered by biosensors over traditional immunoassay screening methods with respect to food analysis, include automation, improved reproducibility, speed and real time analysis. The miniaturisation of immunoassays and biosensors towards nanosensing offers enhanced sensitivity, portability and multiplexing capabilities. Increased demands from stakeholders and consumers to improve food integrity illustrate a need for new tools including smart nano-technologies for sample preparation and analysis. The aim of this work is to show the progress that has been made in the development and validation of nanoarrays as next generation lateral flow arrays to be fit for purpose for the detection of both single and multiple contaminants in environmental and food samples to offer an interchangeable and holistic approach to agri-food safety. ELISA spot and planar waveguide technologies have been developed for the rapid and multiplex analysis of marine biotoxins, allergens, antibiotic residues, pesticides and mycotoxins to be compatible with food control procedures. Combining advances in sample preparation tools, portable nanosensors and remote connectivity offer solutions for improved food security.

**Keywords:** detection system; natural toxin; SMART sensor; toxin

### 5.6. Characterisation of the First Animal Toxin Acting as an Antagonist on AT1 Receptor

BaelenAnne-Cécile Van*IturriozXavierChaigneauMarionKesslerPascalGillesNicolasServentDenisRobinPhilippeUniversité Paris-Saclay, CEA de Saclay, Joliot, DMTS, SIMoS, EMR CNRS/CEA 9004, Gif-sur-Yvette, France.*Correspondence: anne-cecile.van-baelen@universite-paris-saclay.fr

**Abstract:** Peptide toxins from venoms display multiple biological functions associated with varied chemical and structural properties (sequence, cysteine pattern and number, post-translational modifications). The renin angiotensin system (RAS) is one of the main systems regulating cardiovascular homeostasis and its overactivation with increase in angiotensin II level is the main cause of arterial hypertension. Angiotensin II and numerous angiotensin-derived peptides act through AT1 and AT2 receptors belonging to the G-protein-coupled receptor (GPCR) family. AT1 is a target of choice for antagonistic molecules for the treatment of hypertension whereas AT2 is still under exploited due to the lack of knowledge of its physiological properties. The discovery of toxins as new ligands of the RAS receptors would be of great interest to better understand the function of these receptors at molecular and physiological levels. We screened a library of 800 toxins on the AT2 receptor by the mean of a radioligand-binding assay and identified one original. A-CTX-cMila is a 7-residues peptide from *Conus miliaris* with only one disulfide bridge displaying micromolar range affinities on AT2 and submicromolar affinity on AT1 receptor. Using various in vitro assays, we were able to determine its pharmacological profile on AT1. The toxin is a competitive antagonist, blocking Gαq, Gαi3, GαoA pathways, β-arrestin2 recruitment and the activation of ERK. This toxin, with its interesting structural and pharmacological profiles, may represent promising tool for the study of angiotensin receptors. More investigations are needed to fully characterize this toxin, especially on AT2 receptor function. These results describe not only the first animal toxin active on angiotensin receptors, but also the first toxin acting as an antagonist on AT1 receptor.

**Keywords:** AT1; BRET; conotoxin; HTRF

### 5.7. Towards a High-Throughput Mass Spectrometry Methodology to Fish Peptide Toxins from Crude Venoms by Affinity Capture on Cell Membrane Receptors

FreuvilleLou^1^*AmorimFernanda Gobbi^1^VitelloRomain^2^FourmyRudy^3^VioletteAude^3^LiegeoisJean-François^2^BransAlain^4^QuintonLoïc^1^1Mass Spectrometry Laboratory, MolSys Research Unit, Department of Chemistry, University of Liège, allée du Six Août, 11—Quartier Agora, Liège, Belgium.2Laboratory of Medicinal Chemistry and C.I.R.M, University of Liège, Liège, Belgium.3Alphabiotoxine Laboratory sprl, Montroeul-au-bois, Belgium.4Centre for Protein Engineering, University of Liège, Liège, Belgium.*Correspondence: lfreuville@uliege.be

**Abstract:** Animal venoms are complex chemical cocktails, containing a wide range of biologically active peptides whose selectivity and efficiently act against membrane targets, such as ion channels. Venom peptides are largely used in pharmaceutical domains. Toxin targets are involved in various human pathologies such as cancer, neurodegenerative diseases and depression. The scope of venoms for drug discovery is rapidly emerging but is still mostly undone, mainly due to the low availability of compounds, the species size and the complexity of venoms, but also due to the low throughput observed when venoms have to be screened towards various molecular receptors of interest. In this context, our work aims to propose a faster methodology of toxin selection based on affinity capture on cellular membranes and monitored by mass spectrometry. To reach this goal, a set of ten arthropod species was selected due to their high content of toxins potentially targeting potassium channels, which are one of our receptors of interest. The venoms were firstly analyzed by shotgun proteomics (Q-Exactive), following the classical bottom-up approach (reduction, alkylation and digestion with trypsin). The sequences of peptides obtained by digestion were compared to toxins already known to be active on potassium channels. Cellular membranes overexpressing potassium channels were produced, purified and prepared in-house, from transfected HEK293 cells. The receptor concentrations were measured with saturation binding experiments, where membranes were incubated with increasing concentrations of radioligand. Cellular membranes were incubated together with chromatographic fractions of venoms. Toxins displaying affinities for the overexpressed ion channels bind to the membranes, whereas toxins that do not display any affinity are maintained in solution. The mixture containing the candidates is analyzed by MALDI mass spectrometry to identify the potential ligands. This study aims at enables us to highlight potential new toxins targeting potassium channels with a high-throughput filtration method. 

**Keywords:** arthropod; ion channel; toxin screening

### 5.8. Snake Venom Proteins vs ADDobodies—Antigen Production for Ribosome Display

Boldrini-FrançaJohara^1^,^2^*StennerRichard^1^,^2^*BergerImre^1^,^2^Berger-SchaffitzelChristiane^1^,^2^1School of Biochemistry, University of Bristol, 1 Tankard’s Close, Bristol BS8 1TD, United Kingdom.2Bristol Synthetic Biology Centre BrisSynBio, 24 Tyndall Ave, Bristol BS8 1TQ, United Kingdom.*Correspondence: johara.stringari@bristol.ac.uk; rs15090@bristol.ac.uk

**Abstract:** Aim- Snakebite envenoming is a Neglected Tropical Disease that annually causes up to 138,000 deaths and 400,000 disabilities in surviving victims. Current anti-venoms, manufactured from hyperimmunised animals, are weakly effective as only 10–15% of the pooled anti-venom neutralizes toxins. The ADDovenom project aims to use state-of-the-art protein engineering and display technology to generate novel, high-affinity, and safe anti-venoms from naïve libraries. Methods- Ribosome Display is an in vitro selection and evolution method for generating high-affinity antibodies from naïve libraries. Using our nanobody and ADDobody libraries, ribosome display will be used to generate high-affinity binders against a diverse range of snake venom antigens. The high-affinity binders will be isolated, biophysically characterized, and their efficacy evaluated in cell-based assays at the Liverpool School of Tropical Medicine (in collaboration with Prof. Nicholas Casewell). The antigens used in the selections include recombinant venom proteins expressed from bacterial expression systems (in collaboration with Dr. Renaud Vincentelli) and MultiBac insect expression systems, and the EpiString constructs developed by Prof. Robert Harrison. Results- To date, multiple *Echis* phospholipase A2s (PLA2s) and a hyaluronidase have been recombinantly expressed from insect cells, and multiple *Echis* disintegrins and metalloprotease EpiStrings have been recombinantly produced from bacteria. The activities of all the antigens have been confirm. All the antigens have been biotinylated prior to ribosome display, with this modification having no deleterious effect on activity. An ADDobody library has been generated in a format suitable for ribosome display and is actively being employed in selections to generate high-affinity binders against purified PLA2s, disintegrins, and hyaluronidase. Initial rounds of ribosome display, using the ADDobody library, have already yielded results against the aforementioned targets, especially PLA2s and hyaluronidase. In addition, a naïve nanobody library has also been generated (by Dr Huan Sun and Dr Johara Stringari). Main Conclusions- The primary goal of the ADDovenom project is to combat snakebite envenomation by producing safe, effective, and affordable antivenoms. The preliminary data indicate that ribosome display is an effective methodology for accomplishing this objective by generating high-affinity binders against an assortment of pertinent snake venom targets.

**Keywords:** ADDobody anti-venom; recombinant snake venom; ribosome-display

### 5.9. The Venom of the Lebanese Viper Montivipera bornmuelleri Exhibits Neuro- and Cardiovascular Toxicity on Zebrafish 

SahyounChristina^1^,^2^*MatteiCésar^1^LegrosChristian^1^FajlounZiad^2^,^3^RimaMohamad^2^,^4^1University of Angers, MITOVASC, Equipe CarMe, SFR ICAT, INSERM, CNRS, Angers, France.2Lebanese University, Laboratory of Applied Biotechnology (LBA3B), Azm Center for Research in Biotechnology and Its Applications, Tripoli, Lebanon.3Lebanese University, Department of Biology, Faculty of Sciences 3, Tripoli, Lebanon.4Université de Strasbourg, Institut de Génétique et de Biologie Moléculaire et Cellulaire (IGBMC), INSERM, CNRS, Illkirch, France.*Correspondence: christina.sahyoun1@gmail.com

**Abstract:***Montivipera bornmuelleri* is a Viperidae snake endemic to high altitudes of the Lebanese mountains. This snake is listed as endangered by the IUCN, and its venom is poorly characterized. Snakes of the Viperidae family usually possess hemotoxic venoms. This agrees with our previous study showing that *M. bornmuelleri* venom exhibits pro- and anti-coagulant effects supporting its hemotoxic nature. To date, only one human case was reported for an envenomation by *M. bornmuelleri*. A 31-year-old healthy man was bitten in his right leg and developed generalized tonic-clonic seizures shortly. He also developed long-term neurological symptoms such as a right lower limb motor weakness, complete sensory deficit in the groin area, permanent bladder areflexia and an erectile dysfunction suggesting the potential neurotoxicity of this venom. Thus, the aim of our research was to evaluate the putative neurotoxic effects of this venom. We used the zebrafish model to observe the neurotoxicity of the venom. First, *M. bornmuelleri* venom exhibited toxicity to zebrafish embryos since it decreased their viability in a dose dependent manner with an LC50 of ~62 µg/mL. Notably, the venom induced developmental toxicity but not teratogenicity. *M. bornmuelleri* venom also increased zebrafish tail coiling at 1 mg/mL indicating its neurotoxic effects. At the same concentration, the venom disrupted the zebrafish cardiovascular system, leading to heartbeat rate reduction and hemorrhage. Moreover, the venom induced cytotoxicity in vitro on neuronal cultures at doses ranging between 10^−3^ and 10^−1^ mg/mL (P19-derived neurons and primary cortical neurons). Altogether, our findings demonstrate that *M. bornmuelleri* venom displays toxicity against the nervous and cardiovascular systems, suggesting that it contains various toxins responsible of these effects.

**Keywords:***Montivipera bornmuelleri*; neurotoxicity; zebrafish

### 5.10. Immobilisation of Neuro-2a Cells on Electrodes and Electrochemical Detection of MTT Formazan Crystals to Assess Their Viability

AlkassarMounira*LeonardoSandraDiogèneJorgeCampàsMònicaInstitute of Agrifood Research and Technology (IRTA), La Ràpita, Spain.*Correspondence: mounira.alkassar@irta.cat

**Abstract:** Marine neurotoxins are natural products, some of them extremely toxic. They enter the food webs, potentially reaching humans and causing intoxications, which can be mild but also severe and even lead to death. Although some marine toxins have been extensively described, studied and legislated, some others are emerging. Examples of emerging marine toxins are ciguatoxins (CTXs), produced by microalgae of the genera *Gambierdiscus* and *Fukuyoa*, and tetrodotoxins (TTXs), produced by bacteria. Those neurotoxins have been demonstrated to bind to and modulate the activity of cell membrane voltage-gated sodium channels (VGSCs). CTXs block these channels in an open state, whilst TTXs block them in a closed state. These toxins usually produce a change in the physiology, the morphology and/or the viability of cells. In Europe, because the emergence of these toxins is a serious concern for public health, the evaluation and characterisation of their risk are priorities for the European Food Safety Authority (EFSA), which also encourages the search for occurrence data and the development of reliable and efficient analytical methods. Among the different methods, electrochemical cell-based biosensors are a promising strategy. They are based on cell-based assays, which measure the toxicological effect of toxins on cells and provide an estimation of the composite toxicity of a sample, and have the added advantage of the high sensitivity provided by electrochemical detection techniques. With this aim, Neuro-2a cells were immobilised on electrodes of different materials, and their viability was assessed using a tetrazolium salt. After optimisation of the assay conditions, the effect of CTX and TTX standard solutions on the immobilised cells was evaluated. Finally, the system was applied to the analysis of fish extracts containing CTX (*Variola louti*) and TTX (*Lagocephalus sceleratus*), as a proof of concept. The strategy has been proved to be successful and therefore paves the way towards the development of electrochemical cell-based biosensors for the detection of marine neurotoxins. 

**Keywords:** ciguatoxin; electrode material; Neuro-2a cell viability; tetrazolium salt; tetrodotoxin

### 5.11. Structural Diversity and Dynamics of the Complex Formed by Calmodulin with the CyaA Regions Translocated into Host Cells

ChenalAlexandre*Biochemistry of Macromolecular Interactions Unit, Department of Structural Biology and Chemistry, Institut Pasteur CNRS UMR3528, Paris, France.*Correspondence: alexandre.chenal@pasteur.fr

**Abstract:***Bordetella pertussis,* the causative agent of whooping cough, secretes an adenylate cyclase toxin (CyaA) that plays an essential role in the early stages of respiratory tract colonization. The cell intoxication process of CyaA is still poorly understood. Our results, based on a combination of biophysical approaches, illustrate how the structural flexibility of CyaA is essential for its secretion, its folding, its translocation across plasma membrane and target cell intoxication. All of these steps involve disorder-to-order structural transitions that are finely tuned to the environmental conditions that CyaA successively experiences along its journey from the bacterium to the eukaryotic cell cytoplasm. After its secretion through a type 1 secretion system, CyaA intoxicates human cells via a direct translocation of its catalytic domain (ACD) across the plasma membrane. We recently showed that the CyaA P454 region (CyaA residues 454-484 from the translocation region, TR) can translocate across membranes and interact with calmodulin (CaM). Trapping of CyaA by the CaM:P454 interaction in the cytosol may assist the entry of ACD by converting the stochastic motion of the polypeptide chain through the membrane into an efficient vectorial chain translocation into host cells. Once in the cytosol, ACD also binds to CaM and catalyses high amounts of cAMP, leading to cell death. Here, we present the structural models of the CyaA translocated regions, i.e., ACD and TR, in interaction with calmodulin. Our integrative structural biology approach deciphers the structural diversity and dynamics of the complex formed by calmodulin and the CyaA translocated regions.

**Keywords:** adenylate cyclase; binding-induced folding; calmodulin; HDX-MS; intrinsically disordered region; protein-protein interactions; protein membrane translocation; protein structure; protein dynamics; SEC-SAXS; structural model

## 6. Poster Presentations

### 6.1. Evidence of Rattlesnake Crotalphin Effects on Tetrodotoxin-Sensitive Na-Channels of Mouse Dorsal Root Ganglion Neurons

AntunesAurélie^1^,^2^*RobinPhilippe^1^MourierGilles^1^WaardMichel De^2^,^3^,^4^BéroudRémy^2^ServentDenis^1^BenoitÉvelyne^1^1Université Paris-Saclay, CEA de Saclay, Joliot, DMTS, SIMoS, EMR CNRS/CEA 9004, Gif-sur-Yvette, France.2Smartox Biotechnology, Saint-Egrève, France.3Institut du thorax, INSERM, CNRS, Université de Nantes, Nantes, France.4LabEx “Ion Channels, Science and Therapeutics”, Valbonne, France.*Correspondence: aurelie.antunes@cea.fr

**Abstract:** Crotalphine is an analgesic peptide identified in the venom of the South American rattlesnake *Crotalus durissus terrificus*. Although its antinociceptive effects are well documented, its direct mechanisms of action are still under investigation. The aim of the present work was to study the action of the crotalid peptide on the sodium channel subtype NaV1.7, a genetically validated pain target. To this purpose, the effects of crotalphine were evaluated on the tetrodotoxin-sensitive Na current of dorsal root ganglion neurons of adult mice, using the whole-cell patch-clamp configuration, and on the cell viability, using the propidium iodide fluorescence and trypan blue assays. The results show that 18.70 µM of peptide were necessary to inhibit 50% of the Na current, the blocking effects occurring without any marked change in the current activation and inactivation kinetics and voltage-dependencies. In addition, a crotalphine-induced increase in leakage current amplitude of approximately 150% and a maximal decrease of 28% of cell viability were detected in the presence of 50 µM of peptide. Taken together, these results point out, for the first time, the effectiveness of crotalphine to act on the NaV1.7 channel subtype, directly and/or indirectly by affecting cell membrane integrity. This mechanism of action could contribute to the peptide analgesic properties.

**Keywords:** crotalphine; electrophysiology; pain

### 6.2. NeuroTorp Paves the Way for the Immobilization of Complex Lipoprotein Vesicles in the Test-Line of Lateral Flow Assays

TrichetMichaël^1^AráozRómulo^2^,^3^*1Sorbonne Université, CNRS, SME, IBPS, 75005 Paris, France.2Université Paris-Saclay, CEA de Saclay, Joliot, DMTS, SIMoS, Laboratoire Toxines, récepteurs et canaux ioniques, 91191 Gif-sur-Yvette, France.3EMR CNRS/CEA 9004, 91191 Gif-sur-Yvette, France.*Correspondence: romulo.araoz@cea.fr

**Abstract:** NeuroTorp lateral flow test (NeuroTorp-LFT) is a functional method based on the mechanism of action of cyclic imine toxins (CiTx), which are potent antagonists of muscle-type nicotinic acetylcholine receptors. Innovative aspects of NeuroTorp device are the use of glass microfiber membranes as stationary phase for LFT and the immobilization of *Torpedo marmorata* electrocyte membranes as a source of nicotinic acetylcholine receptors in the test-line. GF/C glass microfiber filter is an inert porous membrane with low unspecific binding currently used for preparing the conjugate pad that uniformly release the detector reagent into the mobile phase in lateral flow assays. *Torpedo* electrocyte membranes are a validated source of nicotinic receptors of muscle-type. When applied to the sample pad, cyclic imine toxins will compete with the toxin-tracer for the acetylcholine-binding site of nicotinic acetylcholine receptors immobilized on the NeutorTorp-LFT. The degree of inhibition is CiTx-concentration dependent. Scanning electron microscopy studies enabled the elucidation of the mechanism by which *Torpedo* electrocyte membranes are immobilized. Following application, the *Torpedo* electrocyte membrane vesicles are retained in the voids of GF/C microfibers and after collapsing, the membranes anchor onto the neighboring microfibers randomly placed at the same plane forming lamellar membrane structures. The resulting novel nanocomposite interphase resists drying and is stable through the time (years) providing an optimal environment for the interaction of nicotinic ligands with their receptor target. Scanning electron microscopy also corroborated the specific interaction of α-bungarotoxin with the anchored Torpedo-nicotinic acetylcholine receptor. Taking all together, NeuroTorp-LFT paves the way for immobilizing multiprotein complexes and even cellular membranes on the test-line of lateral flow assays rather than a single biological or chemical macromolecule.

Acknowledgements: We thank the NRBC-E Program (MULTITOX project), the INTERREG Atlantic Area (ALERTOX-NET project), the LABEX LERMIT (DETECTNEUROTOX project) and the GDR PHYCOTOX. 

**Keywords:** cyclic imine; lateral flow test; nicotinic acetylcholine receptor

### 6.3. C-Jun N-Terminal Kinase (JNK) Post-Translational Regulation of Pain-Related ASIC1b and ASIC3-Containing Channels Identified by Functional and Toxin-Based Pharmacological In Vitro and In Vivo Approaches

VerkestClément^1^,^2^DiochotSylvie^1^LinguegliaEric^1^BaronAnne^1^*1Université Côte d’Azur, CNRS, Institut de Pharmacologie Moléculaire et Cellulaire (IPMC), LabEx ICST (Laboratory of Excellence “Ion Channel Science and Therapeutics”), FHU InovPain (Fédération Hospitalo-Universitaire “Innovative Solutions in Refractory Chronic Pain”), Sophia Antipolis, France.2Department of Anesthesiology, University Medical Center Hamburg-Eppendorf, 20251 Hamburg, Germany.*Correspondence: anne.baron@ipmc.cnrs.fr

**Abstract:** Acid-sensing ion channels (ASICs) are voltage-independent H^+^-gated cation channels largely expressed in rodents and humans neurons. Six isoforms (ASIC1a, 1b, 2a, 2b, 3 and 4) associate into homo- or hetero-trimeric channels. Several animal venom-derived peptidic toxins were shown to target ASICs, including PcTx1 from *Psalmopoeus cambridgei* tarantula, APETx2 from *Anthopleura elegantissima* sea anemone, mambalgins from *Dendroaspis sp.* mamba snakes, and the dimeric protein MitTx from *Micrurus tener* coral snake, which have been instrumental to study the structure and the pH-dependent gating of rodent and human ASICs, and their physiological and pathological roles. Neuronal ASICs participate in the detection of tissue acidosis, a hallmark of several painful diseases often also involving the activation of various signaling pathways such as the mitogen-activated protein kinases (MAPKs). Here we identify JNK as a new post-translational positive regulator of recombinant ASICs targeting both rodent and human ASIC1b and ASIC3 subunits (except mouse ASIC3). This regulation is lost upon mutation of a putative JNK phosphorylation site within the intracellular N- and the C-terminal domain of the ASIC1b and ASIC3 subunit, respectively. We used a set of specific and potent ASIC-blocking toxins (PcTx1, APETx2, and mambalgin-1) to perform a pharmacological profiling of ASIC-like transient currents recorded in cultured rodent dorsal root ganglia (DRG) neurons and of the ASIC channel subtypes possibly associated, and to systematically test their possible short-term JNK regulation. Our results demonstrate that the vast majority of DRG neurons expressing ASIC currents are positively regulated by JNK activation of ASIC1b- and ASIC3-containing channels, and that this regulation is involved in the rapid potentiation of ASIC current by the pain-related and proinflammatory cytokine TNFα, a well-known activator of receptor-mediated JNK activity. The pathophysiological relevance of JNK regulation of ASICs was tested in vivo on the spontaneous pain behavior induced by subcutaneous intraplantar injection of a pH 5.0 solution in the mouse hindpaw. Local JNK activation induces a short-term potentiation of the acid-induced cutaneous pain in inflammatory conditions that is partially blocked by the ASIC1-specific inhibitor mambalgin-1. We thus identified a new JNK-mediated fast potentiation of ASIC1b- and ASIC3-containing channels that may contribute to peripheral pain sensitization in inflammatory, neuropathic and migraine pain where ASIC1b and/or ASIC3-containing channels have been involved. The JNK regulation is conserved in human channels and interfering with this regulation might be of potential therapeutic benefit against pain.

**Keywords:** APETx2; ASIC; JNK; mambalgin-1; pain; PcTx1; TNFα

### 6.4. Gastrointestinal Tract Ultrastructural Evaluation: Does Okadaic Acid Differentially Affect the Intestine?

CostasCelia^1^LouzaoM. Carmen^1^*Raposo-GarcíaSandra^1^ValeCarmen^1^VieytesMercedes R.^2^BotanaLuis M.^1^1Departamento de Farmacología, Facultad de Veterinaria, Universidad de Santiago de Compostela, Lugo, Spain.2Departamento de Fisiología, Facultad de Veterinaria, Universidad de Santiago de Compostela, Lugo, Spain.*Correspondence: mcarmen.louzao@usc.es

**Abstract:** Diarrhetic Shellfish Poisoning (DSP) is a foodborne illness caused by ingestion of contaminated seafood products. The lipophilic polyether compounds gathered in okadaic acid-group of toxins are responsible for this intoxication, being okadaic acid (OA) the reference toxin. Gastrointestinal signs such as incapacitating diarrhoea, vomiting, nausea and abdominal pain typically appear from 30 min to several hours after consumption. Not only in humans, but also in mice, diarrhoea has been characterised as the most frequent symptom. Pathophysiology of diarrhoea is complex due to the wide array of alterations that can lead to this outcome. OA is an inhibitor of serine/threonine protein phosphatases (PPs), mainly PP1 and PP2A. Their ubiquitous distribution and involvement in the regulation of a wide array of pathways has led to cellular alterations such as cytoskeletal reorganization. We aimed to assess whether OA caused cytomorphological alterations in different regions along the gut at the moment of diarrhoea. OA was given by oral gavage to mice and they were observed closely, since diarrhoea onset was set as the end of the experiment. Samples of duodenum, jejunum, ileum, and proximal and distal colon were evaluated by Transmission Electron Microscopy. Impairment caused by OA was of greater magnitude in the duodenum, whereas it gets gradually milder along the gut. This could be suggesting a faster effect on the proximal end of the intestine triggering the cascade response resulting in diarrhoea.

**Keywords:** diarrhetic shellfish poisoning; intestinal epithelium; okadaic acid

### 6.5. Oxostephanine, Thalmiculine and Thaliphyline, Three Isoquinoleine Alkaloids Which Inhibit L-Type Voltage-Gated Ca^2+^-Channels

FrangiehJacinthe^1^,^2^*LegendreClaire^1^BreardDimitri^3^RichommePascal^3^HenrionDaniel^1^FajlounZiad^2^MatteiCésar^1^RayAnne-Marie Le^3^LegrosChristian^1^1University of Angers, INSERM, CNRS, MITOVASC, Equipe CarME, SFR ICAT, F-49000 Angers, France.2Laboratory of Applied Biotechnology, AZM Centre for Research in Biotechnology and its Application, Doctoral School for Sciences and Technology, Lebanese University, 1300 Tripoli, Lebanon.3University of Angers, SONAS, SFR QUASAV, F-49000 Angers, France.*Correspondence: jacynthefrangieh@gmail.com

**Abstract:** The isoquinoline alkaloids (IAs) represent a large and diverse subfamily of phytochemicals in terms of structures and pharmacological activities, including ion channel inhibition. Several IAs, such as liriodenine (an oxoaporphine) and curine (a bisbenzylisoquinoline (BBIQ), inhibit the L-type voltage-gated Ca^2+^ channels (LTCC). In this study, we aimed to search for new blockers of LTCC, which are therapeutic targets in neurological and cardiovascular diseases. We set up a screening assay using the rat pituitary GH3b6 cell line, which expresses two LTCC isoforms, CaV1.2 and CaV1.3. Both LTCC subtypes can be indirectly activated by KCl concentration elevation or directly by the dihydropyridine (DHP), BAY K8644, leading to an increase in the intracellular Ca^2+^ concentration ([Ca^2+^]i). These Ca^2+^ responses were completely blocked by the selective LTCC DHP inhibitor, nifedipine. Thereby, 16 selected IAs were tested for their ability to inhibit KCl and BAY K8644-induced Ca^2+^ responses. We then identified three new potent LTCC blockers, namely, oxostephanine, thalmiculine and thaliphyline. They inhibited LTCC with IC_50_ values in the micromolar range through interaction to a binding site different to that of dihydropyridines. The two subfamilies of IAs, oxoaporphine with oxostephanine, and BBIQs with both thalmiculine and thaliphyline, constitute interesting pharmacophores for the development of future therapeutic leads for neurological and cardiovascular diseases. 

**Keywords:** Fura-2 fluorescent probe; GH3b6 cell; intracellular Ca^2+^ measurement; isoquinoline alkaloid; L-type voltage-gated Ca^2+^ channel

### 6.6. Optimization of Biomolecule Extraction from the Rhizosphere of Plants in Extreme Environments in Tunisia

HachaniMondher^1^,^2^*BrahimRaoua Ben^1^,^3^KhlifMohamed^1^FouzaiKhaoula^1^AssesNedra^1^RegayaImed^1^,^3^1Higher Institute of Environmental Sciences and Technologies of Borj Cedria, University of Carthage, Amilcar 1054, Tunisia.2Georessources laboratory, Water Researches and Technologies Center, Hammam-Lif 2050, Tunisia.3Laboratory of Extremophile Plants, Centre of Biotechnology of Borj Cedria, B.P. 901, Hammam-Lif 2050, Tunisia.*Correspondence: mondher_hachani@hotmail.com

**Abstract:** Extraction of biomolecules from non-biological materials is a high-priority research area on a national and international scale. Due to the possibility of clay-biomolecule bonds, co-extraction of organic matter and the relatively small amounts of biomolecules present in the soil, separating organic molecules from a mineral matrix presents a significant challenge. The aim of this work is to optimize the extraction of active molecules against the microorganism proliferation from the rhizosphere of endemic plants in the extremophile regions of southern Tunisia. Soil samples were collected from the rhizosphere of an endemic plant in southern Tunisia. A preliminary characterization of the soils was carried out to identify the fraction most likely to contain biomolecules. On the fine fraction of the soils, different extraction techniques were used with different solvent concentrations (water, ACN, MOH, ACN+TFA, MOH+TFA). The presence of the same biomolecules in the different rhizospheres of the same plant species was demonstrated by liquid phase chromatography, proving that these biomolecules are excreted by the plant and play a significant role in the natural defense and adaptation of this species to the extreme conditions of southern Tunisia. 

**Keywords:** active biomolecules; extraction; extremophile region; optimization; rhizosphere; Tunisia

### 6.7. Scorpion Venom Components Modulate Inflammatory Response during a Polymicrobial Sepsis

ZeroutiKhedidjaLaraba-DjebariFatimaHammoudi-TrikiDjelila*USTHB, Faculty of Biological Sciences, Laboratory of Cellular and Molecular Biology, BP 32, El Alia, Bab Ezzouar, 16111, Algiers, Algeria.*Correspondence: hammoudid@gmail.com

**Abstract:** Scorpion venoms peptides are known to modify the behaviour of immune cells and to induce the production of inflammatory and anti-inflammatory mediators; such action may interfere with physiological or pathological states, such as infections. Sepsis is a complex inflammatory disease considered as an uncontrolled infectious state. It is characterized by an imbalance of pro and anti-inflammatory responses, which led to the invasion of the pathogen and the dysfunction of the vital organs. The aim of this work was to investigate the effect of sublethal dose of *Androctonus australis hector* venom on a murine model of polymicrobial sepsis, induced by intraperitoneal injection of faecal preparation. Physiological state (stress and body temperature), inflammatory response in peritoneal cavity (bacterial clearance and leukocyte activation) and histopathological changes in lung were explored in septic mice treated with venom and compared to non-treated infected mice. The obtained results reveal that scorpion venom seems to exert a positive immunomodulatory action in this infectious context. The venom was able to stimulate the rapid recruitment of neutrophils, primordial cells in the anti-infective response, as well as resident peritoneal leukocytes and antimicrobial substances release such as myeloperoxidase and hydrogen peroxide. This immunostimulation is well correlated with a clear decrease of pulmonary damage, an amelioration of physiological parameters and effective bacterial clearance in the presence of scorpion venom. This finding can be disclosed as promising approach to control local inflammation and to stimulate anti-infective immune response. However, further studies are required to expand and to determine cellular and molecular mechanism of this modulation.

**Keywords:** immunomodulatory effect; inflammatory response; polymicrobial sepsis; scorpion venom

### 6.8. Metabolomic and Transcriptomic Characterization of the Ability to Produce PST in Alexandrium minutum Using Recombinant Progeny

MaryLou^1^*QuereJulien^1^LatimierMarie^1^SavarVéronique^2^HégaretHélène^3^ArtigaudSébastien^3^RéveillonDamien^2^GacMickael Le^1^1Ifremer, Dyneco, Pelagos, Plouzané, France.2Ifremer, Phytox, Metalg, Nantes, France.3Laboratoire des Sciences de l’Environnement Marin (LEMAR), UMR 6539 CNRS UBO IRD IFREMER—Institut Universitaire Européen de la Mer, Plouzané, France.*Correspondence: loumaryupmc@gmail.com

**Abstract:** Paralytic Shellfish Toxins (PST) are derived from the secondary metabolism of certain species of cyanobacteria and dinoflagellates, notably those of the genus *Alexandrium*. In cyanobacteria, these toxins are produced by *sxt* genes, present as a cluster in the genome. Part of the PST biosynthetic pathway has been elucidated in cyanobacteria, and the implication of certain *sxt* genes has been confirmed by experimental studies (heterologous expression, synthesis of intermediates). In dinoflagellates, potential homologs of *sxt* genes have been identified in transcriptomes, and their expression or gene copy number were tentatively linked to the ability to produce toxins. In addition, some biosynthetic intermediates were reported in toxic strains of *A. minutum* and *A. tamarense*. However, there is no study showing the link between sxt genes and metabolites involved in PST synthesis in *A. minutum*.

Therefore, we aimed at understanding the interaction between the genes and the metabolites produced during toxin synthesis, in *A. minutum* strains resulting from a recombinant cross of two toxic x non-toxic parent strains, using a joint non-targeted metabolomic and transcriptomic approach. We were able to confirm that the expression of the *sxtI*, *sxtG* and *sxtA* genes was correlated with the toxic phenotype, as well as a methyltransferase, a transcript with a domain similar to the *sxtS* gene, and other unidentified genes. In addition, we identified a number of metabolites that are strongly involved in toxin production. Among them, a metabolite with a mass-to-charge ratio m/z 236.1139 (-MS), which could not be identified, seems particularly related to this phenotype. Finally, Int-C’, a synthesis intermediate involved in PST synthesis in *A. tamarense* and toxic cyanobacteria, could also be involved in toxin production in *A. minutum*. This is the first study linking genes and metabolites to PST biosynthesis in *A. minutum*. Our results are consistent with parts of the biosynthetic pathway already identified in dinoflagellates and cyanobacteria, but we present new candidates, potentially specific to *A. minutum*.

**Keywords:***Alexandrium minutum*; metabolomics; Paralytic Shellfish Toxins (PST); *sxt* genes; transcriptomics

### 6.9. Evaluation of Clostridium Botulinum A5 Neurotoxin Actions In Vivo and Ex Vivo at the Mouse Skeletal Neuromuscular Junction

BenoitÉvelyne^1^CouesnonAurélie^2^IorgaBogdan I.^3^PopoffMichel R.^4^MolgóJordi^1^,^2^*1Université Paris-Saclay, CEA de Saclay, Joliot, DMTS, SIMoS, EMR CNRS/CEA 9004, Gif-sur-Yvette, France.2Institut des Neurosciences Paris-Saclay, UMR 9197 CNRS/Université Paris-Sud, Gif-sur-Yvette, France.3CNRS, Institut de Chimie des Substances Naturelles, UPR 2301, Labex LERMIT, Gif-sur-Yvette, France.4Institut Pasteur, Unité des Bactéries Anaérobies et Toxines, Paris, France.*Correspondence: jordi.molgo@cea.fr

**Abstract:***Clostridium botulinum* neurotoxins (BoNTs), the most potent toxins known, are the cause of a worldwide life-threatening disease in humans and animals known as botulism. This disease usually manifests as descending symmetrical flaccid paralysis of skeletal muscles together with autonomic dysfunction. BoNTs include a large family of zinc metalloproteases that can be immunologically distinguished by homologous antitoxins into seven primary serotypes, designated A to G. Additionally, for BoNT/A, at least 8 subtypes (/A1-/A8) have been identified from gene sequence analysis. In France, several cases of human botulism due to BoNT/A5, /A6 or /A7 have been reported, though BoNT/A1 and /A2 are the prevalent forms of type-A botulism. The characterization of the *C. botulinum* strains involved in two cases of BoNT/A5-poisoning revealed that they possess the gene encoding for BoNT/A5 identical to the one previously reported. In the present study, we produced the BoNT/A5 crude complex (3 x 107 LD50/mg) from the *C. botulinum* A5 strain 126.07 [1], and studied in vivo and ex vivo, in murine models, the skeletal neuromuscular block caused by BoNT/A5, at different times after a single local toxin injection into the hind-limbs. The results show that the duration and degree of paralysis depended on the dose of BoNT/A5 and on the mouse strain studied. The transgenic Thy-1-YFP-16 black C57BL6 mice [2] were more sensitive to the action of the toxin than Swiss mice, as revealed by the digit abduction score (DAS) assay and by compound muscle action potential (CMAP) recordings from the same mouse in vivo, at different times after toxin injection. Functional and morphological ex vivo studies on muscles locally injected with BoNT/A5 in vivo reveal (a) the presence of axonal outgrowths (nodal and nerve terminal sprouts), (b) an extension of nicotinic acetylcholine receptor clusters, (c) a reduction of both muscle weight and muscle fiber cross sectional area, and (d) the prolonged atrophy of muscle fibers. Pre- and post-synaptic remodeling was completely abolished by an immune-purified rabbit polyclonal antibody directed against the BoNT/A1-Heavy chain (HcA1), when injected together with BoNT/A5. In conclusion, our results show that the actions of BoNT/A5 have many similarities to those of BoNT/A1 previously reported, although no direct comparison was performed in the present study. BoNT/A5 differed from BoNT/A1 by 5 identified amino acids in the Light chain domain (Lc) and by 32 amino acids in the Heavy chain domain (Hc). The fact that the antibody directed to the HcA1 completely prevented the lethal effect of BoNT/A5, as well as the block of the CMAP *in vivo*, and the morphological changes induced by the neurotoxin suggest that the antibody blocks the entry of BoNT/A5 into motor nerve terminals of the neuromuscular junction.

Acknowledgements. This study was supported in part by the project ANTIBOTAB funded by the European Union, and in part by the CNRS and the Institut Pasteur of Paris. We thank Mrs. Patricia Villeneuve for essential help with the logistics and maintenance of animals at the CNRS animal house facility in Gif-sur-Yvette. 

**References**

1. Mazuet C. et al. Genome Biol. Evol. 2016. 8, 1643-1660. doi: 10.1093/gbe/evw101.
2. Feng G. et al. Neuron. 2000. 28, 41-51. doi: 10.1016/s0896-6273(00)00084-2.

**Keywords**: *Clostridium botulinum* type A5 neurotoxin; compound muscle action potential; muscle atrophy; nerve sprouting; nicotinic acetylcholine receptors distribution; skeletal neuromuscular junction; synaptic remodeling.

### 6.10. Optimization of Antimicrobial Peptide Extraction Methods from Tunisian Date Pits

BrahimRaoua Ben^1^,^2^TliliSofiene^3^FouzaiKhaoula^1^,^5^AounallahFarah^4^AmaraSirine^4^AssesNedra^4^RegayaImed^1^,^4^*1Laboratory of Extremophile Plants, Centre of Biotechnology of Borj Cedria, B.P. 901, Hammam-Lif 2050, Tunisia.2Higher Institute of Biotechnology of Monastir, University of Monastir, Monastir 5000, Tunisia.3Cnam-Intechmer—EPN 8/ LUSAC EA4253 Université de Caen, Bvd de Collignon—Tourlaville 50110 Cherbourg en Cotentin, France.4Higher Institute of Environmental Sciences and Technologies, Borj-Cédria, University of Carthage, Tunis, Amilcar 1054, Tunisia.5Department of Biology, Faculty of Sciences of Bizerte, Carthage University, Bizerte 7021, Tunisia.*Correspondence: imed_regaya@yahoo.fr

**Abstract:** Date pits are a very useful natural waste because they can be transformed into different products like coffee, oil, food supplement powder and even soap (Mrabet et al., 2020). This material is also used in the “khol” or eye-liner preparation, since the ancient Egyptian civilization (Pharanian) until now, in various oriental countries. This product, traditionally known for its therapeutic effect in some eye infections, allowed us to think that it could contain antimicrobial molecules. In bibliography, these molecules are naturally present in different seeds to protect and preserve the embryo, in order to reproduce a new plant. Thus, several methods of molecular extraction from the bibliography (applied to the pits or seeds of other plants) were tested on the pits of the Tunisian “Deglet Nour” dates. Only two extraction solvents were selected to only target the extraction of antimicrobial peptides (AMPs): methanol and acetic acid solvents. Methanolic extracts showed very limited or almost no antimicrobial activity against the tested strains, while extracts obtained from acetic acid showed antimicrobial activity against *Salmonella enterica* ATCC 14028, *Staphylococcus aureus* ATCC 6538, *Bacillus subtilis* ATCC 6633 and *Bacillus pumilus* ATCC 14884. This result demonstrates the presence of antimicrobial agents in this extract. These molecules will be isolated and characterized in order to know their exact chemical structures.

Mrabet A, Jiménez-Araujo A, Guillén-Bejarano R, Rodríguez-Arcos R, Sindic M (2020) Date seeds: a promising source of oil with functional properties. Foods 9:787.

**Keywords:** AMPs; chemical extraction; Tunisian date

### 6.11. Partial Agonist Activity of Neonicotinoids on Rat Nicotinic Receptors: Consequences over Epinephrine Secretion and In Vivo Blood Pressure

ParkJoohee^1^TalyAntoine^2^BourreauJennifer^1^NardiFrédéric De^1^LegendreClaire^1^HenrionDaniel^1^GuérineauNathalie C.^1^,^3^LegrosChristian^1^MatteiCésar^1^Tricoire-LeignelHélène^1^*1University of Angers, INSERM U1083, CNRS UMR 6015, MITOVASC, SFR ICAT, 49000 Angers, France.2Theoretical Biochemistry Laboratory, Institute of Physico-Chemical Biology, CNRS UPR 9080, University of Paris Diderot Sorbonne Paris Cité, 75005 Paris, France.3IGF, University of Montpellier, CNRS, INSERM, 34000 Montpellier, France.*Correspondence: helene.tricoire-leignel@univ-angers.fr

**Abstract:** Nicotine-derived insecticide neonicotinoid exert acute neurotoxic effects by activating nicotinic acetylcholine receptors (nAChRs) in insects but their effects on mammalian nervous system are faintly known. Cholinergic synapses in mammals are crucial for the control of vascular tone, blood pressure and skeletal muscle contraction. We therefore hypothesized that neonicotinoids could affect cholinergic networks in mammals and sought to highlight functional consequences of acute intoxication in rats with sub-lethal concentrations of the highly used acetamiprid (ACE) and clothianidin (CLO). Based on electrophysiological data, we demonstrated that both molecules exhibited a weak agonist effect on rat α3β4 receptors predominantly expressed in ganglia of the vegetative nervous system and the adrenal medulla. Accordingly, 100 and 500 µM neonicotinoids stimulate epinephrine secretion from rat adrenal glands. Challenging ACE or CLO together with nicotine (NIC) ended up with paradoxical effects on secretion. In addition, we measured the rat arterial blood pressure (ABP) in vivo by arterial catheterization. As expected, NIC induced a significant increase in ABP while ACE and CLO did not affect the ABP in the same conditions. However, simultaneous exposure of rats to both NIC and ACE/CLO promoted an increase of ABP and induced a biphasic response, which was unexpected. Modeling the interaction of ACE or CLO on α3β4 nAChR is consistent with a binding site located in the agonist pocket of the receptor. This transversal experimental approach of mammal intoxication with neonicotinoids at different scales, including in vitro, ex vivo, in vivo and in silico, paves the way of the acute and chronic toxicity for this class of insecticides on mammalian organisms.

**Keywords:** arterial blood pressure; catecholamine secretion; neonicotinoid insecticide; nicotinic receptor

### 6.12. Characterisation of the First Animal Toxin Acting as an Antagonist on AT1 Receptor

BaelenAnne-Cécile Van*IturriozXavierChaigneauMarionKesslerPascalGillesNicolasServentDenisRobinPhilippeUniversité Paris-Saclay, CEA de Saclay, Joliot, DMTS, SIMoS, EMR CNRS/CEA 9004, Gif-sur-Yvette, France.*Correspondence: anne-cecile.van-baelen@universite-paris-saclay.fr

**Abstract:** Please refer to section 5.6.

### 6.13. Venomous Snakebites in the Western Lowland of Albania

VrenoziBlerina*University of Tirana, Faculty of Natural Sciences, Research Center of Flora and Fauna, Petro Nini Luarasi, No 80, 1010 Tirana, Albania.*Correspondence: bvrenozi@gmail.com; blerina.vrenozi@fshn.edu.al

**Abstract:** Venomous snakes encountered in Albania belong to the family Viperidae, with three species *V. ammodytes* (Linnaeus, 1758), *V. berus* (Linnaeus, 1758), and *V. ursinii* (Bonaparte, 1835). Snakebites of *V. ammodytes* are known as the most venomous snakes in Europe, and in Albania, this viper is mostly encountered and abundant in the western and southwestern lowlands, in the altitudes 0–1800 m a.s.l. The venom of *V. ammodytes* has higher toxicity showing strong neurotoxicity due to the presence of ammodytoxin-like proteins. Although there are rarely fatal cases, snakebites are medically important in Albania, mainly in the summer season when the fieldwork is carried out during the feeding time of the vipers, out of the midday high temperatures. Therefore, this retrospective study will highlight the importance of snakebite envenoming and raise awareness to prevent their morbidity. The description of the 47 snakebites case studies over a decade, from the villages of Fier County, hospitalized in the regional hospital, is presented. Detailed data on the localities, age, gender, bitten location, supportive treatment, bite date, and hospitalized time, are analyzed. The snakebites were during the seasonal fieldwork, from March to November. Snakes were identified as *Vipera* sp., or simply as snakebite. The hospitalized patients from the snakebites were all farmers from the agricultural areas in Western Lowland. Based on the fieldwork, the venom severity is higher in the summer, due to the high temperatures starting from the last week of May till the first week of September (74.47%). The snakebite localities were particularly the lower and upper limbs of the age groups of 15 to more than 44 years old, although there were also kids helping their parents (8.5%). Almost all patients got the antivenom of *Vipera* sp. (95.74%), except two cases of >44 years old which got alternative treatment. Patients were discharged mainly after 72 h (89.36%), although 6.3% of the case studies had more than three days of hospitalization, and two of them recovered soon after the antivenom injection. The morbidity of the snakebite venom in Albania raises awareness to consider the distribution map of the venomous vipers and take precautions to prevent it.

**Keywords:** Albanian; Balkans; high temperature; snake; summer; venom toxicity; *Vipera ammodytes*

### 6.14. The Synergistic Action of PLA2 and Melittin of Apis Mellifera Syriaca Venom on Colon Cancer Cells HCT116

YaacoubCarole^1^,^2^*El-ObeidDany^3^CoutardBruno^2^FajlounZiad^1^,^4^1Laboratory of Applied Biotechnology (LBA3B), Azm Center for Research in Biotechnology and its Applications, EDST, Lebanese University, 1300 Tripoli, Lebanon.2Unité des Virus Emergents, Aix-Marseille University-IRD 190-Inserm 1207, IHU Méditerranée Infection, Marseille, France.3Faculty of Agriculture & Veterinary Sciences, Lebanese University, Dekwaneh, Beirut 2832, Lebanon.4Faculty of Sciences 3, Lebanese University, Michel Slayman Campus, 1352 Ras Maska-Tripoli, Lebanon.*Correspondence: caroleyaacoub3@hotmail.com

**Abstract:** Colon carcinogenesis is ranked second globally among human diseases after cardiovascular failures. Bee venom (BV) has been shown to display in vitro anticancer effects against several types of cancer cells. The two main biopeptides of *Apis mellifera* BV, namely, melittin (MEL) and phospholipase A2 (PLA2), are suspected to be the biomolecules responsible for the anticancer activity. The present work aims to evaluate the cytotoxic effect of the *A. mellifera syriaca* venom on human colon carcinoma cells (HCT116), and to assess the synergistic effect of MEL and PLA2 on these cells. After analyzing, through high-pressure liquid chromatography, the proportions of MEL and PLA2 on BV, we have established a cell viability assay to evaluate the effect of BV, MEL, PLA2, and a mixture of MEL and PLA2 on the HCT116 cells. Results obtained showed a strong cytotoxicity effect induced by the *A. mellifera syriaca* venom and to a lower extent MEL or PLA2 alone. Remarkably, when MEL and PLA2 were added together, their cytotoxic effect was greatly improved, suggesting a synergistic activity on HCT116 cells. These findings confirm the cytotoxic effect of the *A. mellifera* venom and highlight the presence of synergistic potential activities between MEL and PLA2, possibly inducing membrane disruption of HCT116 cancer cells. Altogether, these results could serve as a basis for the development of new anticancer treatments.

**Keywords:***Apis mellifera syriaca*; bee venom; HCT116 cell line; in vitro anticancer effect; melittin; PLA2

### 6.15. Study of the Effect of Paracetamol on a Model of Bioaccumulation of Toxicity, the Snail Helix aspersa

ZouaghiMohamad Fateh^1^,^2^*TebboubLamia^1^TebboubKawter^1^BoulahdidAbir^1^1Université de Jijel, Algeria.2Laboratoire de Toxicologie Cellulaire, Université de Annaba, Algeria.*Correspondence: zouaghifateh@hotmail.com

**Abstract:** In this study, we were interested in evaluating the effect of a drug, paracetamol, on a model organism for the accumulation of pollution, the snail *Helix aspersa*. This is a subchronic toxicity study (15 days). The toxicity of paracetamol was determined in the snail using a biotest carried out in the laboratory on animals exposed to increasing concentrations of paracetamol (500 mg, 1000 mg, 1500 mg in food). Our results highlight physiological disturbances concerning the weight and the shell diameter of the treated snails. At the same time, the metabolic changes indicate a dose-dependent decrease in the level of proteins and carbohydrates in the hepatopancreas and the kidney. In addition, induction of catalase activity has been demonstrated. The latter constitutes a means of cellular defense against the presence of paracetamol at the level of two target organs.

**Keywords:** bioaccumulator; *Helix aspersa*; paracetamol; toxicity

## 7. Conclusions

The 28th Meeting on Toxinology was held in person with over 80 participants. This event allowed experts in the field of toxinology to “truly” meet again after 2 years (cancelled meeting in 2020 and virtual meeting in 2021), to discuss recent outcomes and future research trends, and to foster or strengthen collaborations. Remarkably, the short lectures delivered by the young researchers and students were of high quality, and they have been well appreciated by all participants, as evidenced by the close scores from the votes for the “Best Oral Communication” and “Best Poster” awards funded by *Toxins*. The recent advances in the finding of new toxins thanks to artificial intelligence; in their detection using biosensors; and in their evolution, genomic or structural analysis will attract new communications in the form of articles and reviews in this Special Issue, to complement the meeting abstracts and to provide greater visibility in the field of toxinology.

## Data Availability

Not applicable.

